# Detection of colorectal lesions during colonoscopy

**DOI:** 10.1002/deo2.68

**Published:** 2021-11-02

**Authors:** Hiroaki Ikematsu, Tatsuro Murano, Kensuke Shinmura

**Affiliations:** ^1^ Division of Science and Technology for Endoscopy Exploratory Oncology Research & Clinical Trial Center National Cancer Center Chiba Japan; ^2^ Department of Gastroenterology and Endoscopy National Cancer Center Hospital East Chiba Japan

**Keywords:** artificial intelligence, blind spot, colorectal lesions, image‐enhanced endoscopy

## Abstract

Owing to its high mortality rate, the prevention of colorectal cancer is of particular importance. The resection of colorectal polyps is reported to drastically reduce colorectal cancer mortality, and examination by endoscopists who had a high adenoma detection rate was found to lower the risk of colorectal cancer, highlighting the importance of identifying lesions. Various devices, imaging techniques, and diagnostic tools aimed at reducing the rate of missed lesions have therefore been developed to improve detection. The distal attachments and devices for improving the endoscopic view angle are intended to help avoid missing blind spots such as folds and flexures in the colon, whereas the imaging techniques represented by image‐enhanced endoscopy contribute to improving lesion visibility. Recent advances in artificial intelligence‐supported detection systems are expected to supplement an endoscopist's eye through the instant diagnosis of the lesions displayed on the monitor. In this review, we provide an outline of each tool and assess its impact on the reduction in the incidence of missed colorectal polyps by summarizing previous clinical research and meta‐analyses. Although useful, the many devices, image‐enhanced endoscopy, and artificial intelligence tools exhibited various limitations. Integrating these tools can improve their shortcomings. Combining artificial intelligence‐based diagnoses with wide‐angle image‐enhanced endoscopy may be particularly useful. Thus, we hope that such tools will be available in the near future.

## INTRODUCTION

Colorectal cancer has a high mortality rate worldwide; thus, its prevention is important. Resecting colorectal polyps found during colonoscopy was reported to reduce colorectal cancer mortality by 53%,^1^ and examinations by endoscopists with a high adenoma detection rate (ADR) have been found to lower the risk of colorectal cancer.^2^ It is therefore important to conduct diagnostic examinations to avoid missing lesions. Nevertheless, although remarkable advancements in endoscopic imaging have enabled high‐resolution observations, it was reported that approximately 25% of neoplastic lesions are missed during a single colonoscopy.^3^, ^4^, ^5^ This situation, therefore, needs to be addressed.

Lesions are typically missed due to the skill of the endoscopist, as well as bowel preparation status; however, there are additional factors associated with the shape and anatomy of the lesions. Blind spots (such as behind folds and flexures) are easily missed, as are flat or depressed lesions (reported to be highly malignant).^6^, ^7^, ^8^, ^9^ Additionally, missing lesions can prove fatal. These factors cannot be addressed using better skills or pretreatment. Therefore, many devices, images, and artificial intelligence (AI)‐based diagnostic tools have been developed recently to improve these areas. Here, we review how these tools can help reduce the incidence of missed colorectal polyps.

### Missing blind spots

The viewing angles of current colonoscopes are limited to 180° or less. Inevitably, with its many folds and flexures, there are blind spots in the large intestine. Retroflexed views (RVs), attachments, wide‐angle observations, and other tools have therefore been developed to avoid missing blind spots, and there have been many reports on their usefulness.

### Usefulness of repeated observations on the right side of the colon

We reviewed repeated observations without an endoscopic device on the right side of the colon. Various studies have shown that colonoscopic observation of the proximal colon is less effective than that of the distal colon for the prevention of colorectal cancer development and death.^10^, ^11^, ^12^ This is because neoplastic lesions in the proximal colon are hidden behind the haustral folds of the colon or anatomic flexures and are thus difficult to detect.^13^ Additionally, the proportions of small neoplastic and flat or depressed lesions—which are also difficult to detect—are higher in the proximal than in the distal colon.^14^ Therefore, the proximal colon needs to be carefully examined to detect neoplastic lesions.

A common endoscopic observation method is the RV of the proximal colon. Retroflexion is often performed from the cecum to the hepatic flexure, often defined as the right side of the colon.^13^, ^15^ An RV of the right side of the colon allows observation from the oral side of the folds, which is known to increase the ADR when combined with the forward view (FV).^15^, ^16^, ^17^ Conversely, a systematic review comparing the adenoma miss rates (AMRs) after observing the initial FV, second FV, and RV reported that the AMR of the initial FV was 7.3% versus 6.3% compared with both the second FV and RV on the right side of the colon; there were no statistically significant differences (*p* = 0.21). In addition, repeated observation (initial + second FV) of the right side of the colon increased right‐sided ADR by 10% (second vs. initial, 33.6% vs. 26.7%), while using the RV on the right side of the colon increased the right‐sided ADR by 6% (RV vs. initial FV, 28.4% vs. 22.7%).^18^

The reason for the lack of difference between the second FV and RV may be as follows. As the shape of the folds and distensions of the right colon change during the initial FV, undetected polyps enter the endoscopist's view upon the second examination, regardless of the direction of the examination.^13^ Still, the addition of a second FV or RV may increase the ADR. Thus, these methods should be considered on the right side of the colon.

In addition to the ascending colon, the rectum is a common site for the RV during colonoscopy. Saad et al. reported that in 1411 cases in which both FV and rectal RV were available, only one neoplastic lesion could be visualized using the RV alone.^19^ Other studies similarly reported that the detection rate of neoplastic lesions that could only be seen on the RV was 0.4%–0.8%.^20^, ^21^ Téllez‐Ávila et al. concluded that although the detection rate of neoplastic lesions was low, the increased possibility of detecting neoplastic lesions that cannot be identified using the FV may make RV suitable.^21^

The limitation of RV on the right side of the colon is that it requires skill in endoscopic manipulation and observation, as it is difficult to manipulate the scope in the RV. Additionally, the shaft of the scope itself covers part of the colon.^16^, ^22^ An RV outside the right colon and rectum is either technically difficult or there are no reports available on its use.

### Usefulness of distal attachment devices

We reviewed the use of distal attachment devices and systems that reduce the incidence of blind spots during colonoscopy. Colonoscopy with a plastic cap (or hood) can facilitate cecal intubation and adenoma detection by maintaining an appropriate distance from the tip of the scope to the mucosa, as well as ensuring a field of view.^23^, ^24^ The device flattens haustral folds to allow for the observation of areas that would otherwise be considered blind spots, such as the folds and anatomic flexures.^25^ It has been reported that cap‐assisted colonoscopy has a higher ADR than standard colonoscopy (SC) without a cap, especially in the right colon or flat adenoma.^24^ However, a meta‐analysis conducted by Ng et al. reported no significant difference in ADR between cap‐assisted colonoscopy and SC (46.8% vs. 45.3%; RR: 1.04; 95% CI: 0.90–1.19).^26^

The Endocuff (Olympus Corporation, Tokyo, Japan) has a fixed portion and two rows of eight soft projections to improve ADR.^27^ The projections fold backward during insertion, which does not interfere with insertion, and are pulled forward to hold back and straighten the colonic folds during withdrawal.^28^, ^29^ Since the Endocuff had the potential to cause colon mucosal abrasions, it was improved and is now available as Endocuff Vision, with a row of longer, softer projections. The ADR when using Endocuff‐ or Endocuff Vision‐assisted colonoscopy (EAC) was reported to be 35%–52%.^28^, ^29^, ^30^, ^31^ In a meta‐analysis conducted by Williet et al. comparing the ADR of EAC and SC, that of EAC was found to be significantly higher (41.3% vs. 34.2%, respectively; RR, 1.20; *p* = 0.003). In another meta‐analysis, the ADR of EAC (which was only performed using Endocuff Vision) was significantly higher than that of SC (49.8% vs. 45.6%, respectively; RR, 1.12; *p* = 0.02).^32^ However, these meta‐analyses were both highly heterogeneous (I^2^ = 79% and 53%, respectively).^32^, ^33^

EndoRings (EndoAid Ltd, Caesarea, Israel) consist of two circular rows of flexible silicone rubber rings. Their structure makes it possible to mechanically straighten the colonic folds, thereby decreasing the number of blind spots and keeping the tip of the colonoscope centered within the colonic lumen during withdrawal.^34^ The ADR of EndoRing‐assisted colonoscopy was reported to be 48.6%–50%.^35^, ^36^ In a meta‐analysis conducted by Facciorusso et al., the ADRs of EndoRing‐assisted colonoscopy and SC were found to be comparable (53.9% vs. 49.1%; RR 1.05, 0.95–1.17).^37^

The G‐EYE (SMART Medical Systems Ltd, Ra'anana, Israel) is a balloon‐assisted colonoscopy (BAC) system that integrates a reusable balloon within the curved portion of a standard colonoscope. The haustral folds and anatomic flexure can be straightened and flattened by withdrawing the scope while inflating the balloon. This system allows for the detection of polyps hidden behind folds and is expected to improve ADRs.^38^, ^39^ The balloon can be inflated up to a maximum diameter of 60 mm, and the internal pressure of the balloon can be controlled via a dedicated inflation system to maintain a constant pressure. In a randomized controlled trial (RCT), BAC provided a significant increase in ADR when compared with SC, with ADRs of 48.0% and 37.5%, respectively (*p* < 0.05).^40^ In the sub‐analysis, BAC identified a significantly higher average number of adenomas per patient than those with SC regarding flat adenomas and sessile serrated lesions, which are said to be difficult to detect on the right side of the colon (flat adenoma: 0.16 vs. 0.05; sessile serrated lesions: 0.04 vs. 0.01; *p* < 0.05).

Although some studies have compared each of these devices, there is no consensus regarding the superiority of any device.^41^, ^42^, ^43^

### Advanced technology in colonoscopy

We reviewed advanced technology aimed at improving ADRs by increasing the endoscopic view angle. The third eye retroscope (TER; Avantis Medical Systems, Inc., San Jose, CA, USA) is an adjunct, through‐the‐scope optical device for detecting polyps behind haustral folds or anatomic flexures.^44^, ^45^ The TER—while extending through the working channel of a standard colonoscope—can automatically retroflex 180° and has a small video camera and light‐emitting diode on its tip. It can therefore provide a continuous retrograde image during withdrawal.^44^, ^46^ Video images obtained using the forward and retrograde views are then displayed side by side on a monitor. Leufkens et al. performed an RCT to evaluate the effect of the TER on ADRs during colonoscopy. They reported that the net ADR of colonoscopy using the TER against SC was 23.2% and concluded that the TER improved the ADR.^47^ The limitation of using the TER is that endoscopists need to become accustomed to simultaneously observing two video images.^48^, ^49^ Additionally, the procedural time may be extended, as the use of a working channel during withdrawal causes problems, such as difficulty with suction and the need to remove the TER for every endoscopic procedure.^48^

**FIGURE 1:**
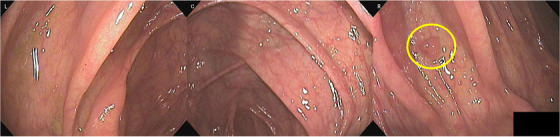
One case using full‐spectrum endoscopy. In the ascending colon, a polyp between the folds is visualized on the right screen

Full‐spectrum endoscopy (FUSE; EndoChoice Inc., Alpharetta, GA, USA) is an endoscopic system with lenses and LEDs mounted on the front and both sides of the scope tip. The FUSE‐colonoscope has an endoscopic right‐left viewing angle of 330° and can simultaneously acquire endoscopic images from three lenses, displaying them on three separate screens. It is expected to reduce the oversight of lesions behind the colonic folds or anatomic flexure by widening the field of view (Figure 1). Gralnek et al. conducted a multicenter, prospective, RCT of standard forward‐viewing colonoscopy (SFVC) versus FUSE.^50^ In this back‐to‐back tandem study—with AMR as the primary endpoint—the AMR of SFVC was 41%, compared with only 7% for FUSE. In Japan, Kudo et al. conducted a similar study.^51^ In this research, the AMR per patient (AMR‐PP) was used as the primary endpoint. The AMR‐PP of SFVC was 22.9%, while that of FUSE was significantly lower, at 11.7% (*p* < 0.05). In the sub‐analysis, they also reported that the AMR‐PP for diminutive lesions and in the ascending colon were both significantly lower with FUSE than with SFVC.^51^ However, the limitation of FUSE is that the endoscopist needs to be comfortable simultaneously looking at three screens. Additionally, while the right‐left viewing angle is considered sufficient, the up‐down viewing angle is narrower than that on the left and right.^52^

One of the goals of endoscopists is to improve the detection rate of colorectal lesions by increasing the endoscopic viewing angle. An extra‐wide‐angle‐view colonoscope (EWAVC) (Olympus Corporation) is therefore currently under development. EWAVC achieves a 235° field of view by constructing a single image from the images obtained by the lens looking forward, as well as the two lenses looking laterally backward. However, this system does not include high‐definition resolution. Uraoka et al. performed a multicenter, single‐arm study using the EWAVC prototype, reporting that 57.1% of adenomatous lesions were detected in the lateral‐backward view before the FV. This tendency was observed in the ascending and sigmoid colons.^53^ In a prospective study, the ADR of EWAVC was 39.9%,^54^ while in an RCT, there were no differences between the ADR of EWAVC and conventional scope with high‐definition resolution (60.3% vs. 59.5%).^55^ As with FUSE, the major limitation is that the endoscopist needs to be accustomed to looking at the screen.^54^ However, the next generation of this system is currently under development.

### Missing lesions that are difficult to recognize

Although polypoid lesions are easy to identify, flat or depressed lesions—such as early‐stage esophageal and gastric lesions—are difficult to identify visually. While using the new tools described above can lead to the enhanced detection of polypoid lesions, they do not improve the visibility of flat and depressed lesions; thus, increasing their detection is difficult. Image‐enhanced endoscopy (IEE) has been reported to be useful for the detection of pharyngeal and esophageal lesions as it improves their visibility.^56^ It was therefore expected that IEE would improve the detection of large intestine lesions, including flat and depressed lesions; indeed, there have been many associated reports.

### Observations with IEE

When IEE was first introduced, many studies used narrow‐band imaging (NBI) and flexible spectral imaging color enhancement.^57^, ^58^, ^59^, ^60^, ^61^, ^62^, ^63^ Most reports were negative trials, and a meta‐analysis by Pasha et al. concluded that neither did NBI increase the detection of colon polyps, adenomas, or flat adenomas nor did it decrease the rate of missed colon polyps or adenomas in patients undergoing screening and surveillance colonoscopy.^64^ Despite reporting a lower rate of missed lesions with NBI than with white‐light imaging (WLI) in an RCT on tandem endoscopy, a significant difference in detection rates during the first observation was not observed.^61^ Flat and depressed lesions tended to be detected more frequently with NBI. Furthermore, in a retrospective study, we found that many flat and depressed lesions could be more easily recognized with IEE than with WLI due to their appearance as brownish areas.^65^

Image darkness is a problem with first‐generation IEEs. Second‐generation IEEs, improving this limitation, have since appeared. Horimatsu et al. and Leung et al. reported significantly higher polyp detection rates and Leung et al., a higher AD—with NBI than with WLI.^66^, ^67^ Additionally, in a meta‐analysis including data from individual patients in RCTs, Atkinson et al. found NBI to have a higher ADR than WLI. This effect was increased when bowel preparation was optimal.^68^ At the same time, blue‐laser imaging (BLI) and linked color imaging (LCI)—which were new IEEs—appeared, with many reports regarding their usefulness.^69^, ^70^, ^71^, ^72^ We reported that the mean number of adenomas found per patient was higher with BLI than with WLI (WLI: 1.01 ± 1.36, BLI: 1.27 ± 1.73; *p* = 0.008),^70^ while Min et al. reported that LCI improved the detection of colorectal polyps and adenomas and was particularly useful for flat lesions.^71^

**FIGURE 2:**
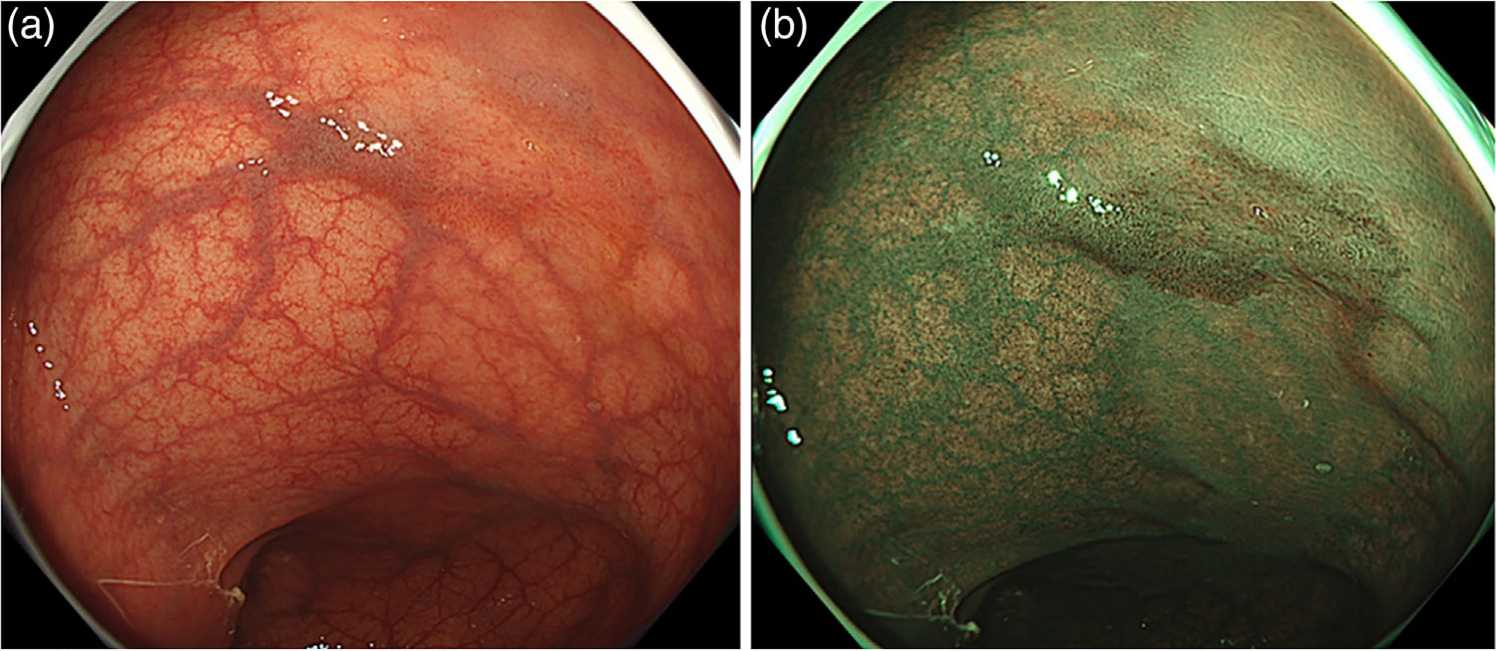
Differences in lesion visualization between white‐light imaging (WLI) and narrow‐band imaging (NBI). The lesion is a 20 mm‐diameter IIa (LST‐NG) lesion in the sigmoid colon. Pathological findings indicate intramucosal cancer. (a) This lesion can be recognized by WLI with faint redness; however, it is difficult to detect. (b) This lesion can be recognized by NBI as a brownish area and is easy to detect

**FIGURE 3:**
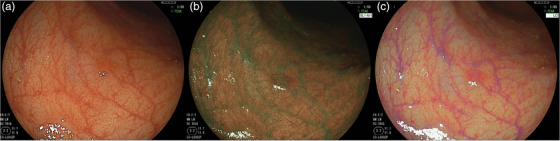
Differences in lesion visualization between white‐light imaging (WLI), blue‐laser imaging (BLI), and linked color imaging (LCI). This lesion is a 5 mm diameter IIc lesion in the sigmoid colon. Pathological findings indicate high‐grade tubular adenoma. (a) This lesion can be recognized by WLI with faint redness; however, it is difficult to detect. (b) This lesion can be recognized by BLI as a brownish line at the edge of the lesion and is easy to detect. (c) This lesion can be recognized by LCI as a pinkish line at the edge of the lesion and is easy to detect

Objective assessment using lesion visibility scoring reported higher levels of visibility for BLI and LCI than for WLI.^73^, ^74^ Furthermore, as additional objective evaluations, we assessed the visibility of colorectal lesions and usefulness of BLI and LCI using eye‐tracking that monitors eye movements. Fewer misses and faster detection were reported with BLI and LCI than with WLI.^75^ BLI and LCI improved the rate of missed colorectal lesions, as well as detection time, compared with WLI. From the above, it was proven that IEE provided better visibility than WLI (Figures 2 and 3). Recently, sessile serrated lesions have received attention due to their difficulty in detection with conventional colonoscopy. Fujimoto et al. reported that LCI was superior to conventional WLI for sessile serrated lesion detection during colonoscopy.^76^ However, there are still few reports on sessile serrated lesion detection, so further consideration is needed in the future.

There have also been reports on another form of IEE—autofluorescence imaging (AFI)—that identifies neoplastic lesions as areas with a magenta color.^77^, ^78^, ^79^ Takeuchi et al. reported a significantly higher ADR using AFI with an attachment than WLI without an attachment.^78^ Additionally, AFI improved the detection of flat colorectal neoplasms in the right‐sided colon compared with WLI.^79^ However, AFI requires a specialized, relatively thick endoscope, limiting its use.

Still, the observation of IEE also has some limitations. First, IEE does not improve the detection of lesions in blind spots. The other is that there may be a learning curve due to the use of unfamiliar images during IEE. We compared the mean number of adenomas per patient—detected using WLI and NBI—in various institutions, including academic and community hospitals.^80^ More lesions were found with NBI at academic institutions, whereas more were detected using WLI at community hospitals. However, a comparison based on the presence or absence of NBI showed that significantly more lesions were found in patients who had undergone NBI than WLI.

### Automatic diagnosis

The detection rate of colorectal lesions has steadily improved with the development of the above‐mentioned manipulation skills during the withdrawal phase, endoscopic instruments, and endoscopic imaging techniques. Nevertheless, it has been reported that skilled endoscopists still miss colorectal lesions during 20% of colonoscopies.^81^ Polyps can be recognized on the monitor for just several seconds during a colonoscopy of several minutes, potentially explaining how endoscopists may overlook the lesions, even if they are displayed on the monitor.

Indeed, several papers demonstrated that the presence of an additional observer—other than an endoscopist—was associated with increased ADRs,^82^, ^83^ highlighting the inevitability of human error in polyp detection during a colonoscopy performed by a single endoscopist. More recently, there have been reports on visual gaze patterns by tracking the eye movement patterns of endoscopists during observation.^84^ It was determined that different endoscopists exhibited different eye movements, and endoscopists with high polyp detection rates were looking around the screen. In other words, depending on the eye movement of the endoscopist, some areas on the screen may not be observed, potentially explaining why lesions are overlooked.

As an innovative solution to this limitation, AI‐based diagnosis supporting the detection of colorectal lesions has attracted a great deal of attention in the past few years. With the advent of deep learning using graphic processing units, the iterative learning of endoscopic images has allowed for AI diagnosis based on the image feature amount of lesions. AI‐supported lesion detection—or computer‐aided polyp detection (CADe)—can alert endoscopists via the instant diagnosis of colorectal lesions that emerge on the monitor. In 2018, Misawa et al. and Urban et al. reported on the performance of AI diagnostic systems using test videos, indicating real‐time polyp detection with over 90% sensitivity.^85^, ^86^ Subsequently, a series of RCTs were conducted to verify the efficacy of CADe in actual clinical settings. Wang et al. provided the first RCT, reporting that the ADR in the CADe group was 29.1%, significantly higher than the control group (20.3%).^87^ Since then, several RCTs from China have been published, reporting the efficacy of CADe methods independently developed by each institution.^88^, ^89^, ^90^, ^91^, ^92^ In addition, Repici et al. examined the efficacy of GI Genius (Medtronic Plc, Dublin, Ireland)—a CADe system designed for clinical implementation without a second monitor—reporting that the ADR in the CADe group was 54.8%, a significant improvement over 40.4% in the control group.^92^ As a result of this achievement, GI Genius received the CE mark in 2019, and the first approval as an AI endoscopic system from the FDA in 2021. Additionally, the DISCOVERY (PENTAX Medical, Tokyo, Japan), EndoBRAIN‐EYE (Olympus Corporation), and CAD‐EYE (Fujifilm, Tokyo, Japan), have all received pharmaceutical approval in both Europe and Japan, suggesting the increased development of similar CADe methods in the future. With the rapid development of CADe, four systematic reviews summarizing the results of RCTs have recently been published.^93^, ^94^, ^95^, ^96^ All reported the superiority of the CADe group over the control group regarding both ADR and polyp detection rate. By contrast, a meta‐analysis reported no difference in the detection of lesions larger than 10 mm, or advanced adenoma, between the CADe and control groups.^93^ The impact of CADe on long‐term clinical outcomes, such as the prevention of mortality by colorectal cancers, is, therefore, a subject for future research. It is also important to examine the efficacy of CADe for detecting frequently missed flat lesions, as well as the positive effects of CADe on ADR when IEE—other than white light—is applied during the withdrawal.

Despite its prominent contribution to the improvement of lesion detection, the limitation of CADe lies in the fact that it can only alert endoscopists when the lesions are displayed on the monitor. Therefore, it remains necessary for endoscopists to carefully observe the entire colon—especially blind spots behind the colon fold or flexion area—using the appropriate endoscopic instrument and manipulation, as discussed above.

Recently, an AI‐ supporting system, WISENSE, was developed to monitor blind spots during Esophagogastroduodenoscopy. This system can give endoscopists the information about the observed sites in a real‐time manner and is reported to reduce the blind spot rate of the Esophagogastroduodenoscopy procedure.^97^ This type of AI‐based quality management system is expected to be further progressed and reduce the blind spot during colonoscopy in near future.

## CONCLUSION

In summary, we reviewed various devices, IEE, and AI tools aimed at preventing blind spots from being missed. While all are considered useful, they also have drawbacks. Integrating these tools can therefore improve their shortcomings. Combining AI diagnosis with wide‐angle IEE may be the most useful technique, and we hope that such tools will be developed in the future.

## CONFLICT OF INTEREST

Hiroaki Ikematsu is an associate editor of DEN Open. The rest of the authors have no conflict of interest.

## FUNDING INFORMATION

None
